# Mishaps and unsafe conditions in recreational scuba diving and pre-dive checklist use: a prospective cohort study

**DOI:** 10.1186/s40621-017-0113-z

**Published:** 2017-06-05

**Authors:** Shabbar I. Ranapurwala, Steve Wing, Charles Poole, Kristen L. Kucera, Stephen W. Marshall, Petar J. Denoble

**Affiliations:** 1 Department of Epidemiology, University of North Carolina, Chapel Hill, NC USA; 2 Divers Alert Network, Durham, NC USA; 3 Department of Exercise and Sports Science, University of North Carolina, Chapel Hill, NC USA; 4 Injury Prevention Research Center, University of North Carolina, Chapel Hill, NC USA

**Keywords:** Scuba diving, Pre-dive checklists, Mishaps, Unsafe conditions, Recreation

## Abstract

**Background:**

Recreational scuba diving involves the use of complex instruments and specialized skills in an unforgiving environment. Errors in dive preparation in such an environment may lead to unsafe conditions, mishaps, injuries and fatalities. Diving mishaps can be major and minor based on their potential to cause injury and the severity of the resulting injury. The objective of this study is to assess the incidence of diving mishaps and unsafe conditions, and their associations with the participants’ routine use of their own checklists.

**Methods:**

Between June and August 2012, 426 divers participated in the control group of a randomized trial to evaluate the effectiveness of an intervention pre-dive checklist. The current nested analysis prospectively follows the control participants, who did not receive the intervention checklist. Poisson regression models with generalized estimating equations were used to estimate rate ratios comparing written checklist use with memorized and no checklist use.

**Results:**

The overall incidence of major mishaps and minor mishaps was 11.2 and 18.2 per 100 dives, respectively. Only 8% participants reported written checklist use, 71% reported using memorized checklists, and 21% did not use any checklist. The rate ratio for written checklist use as compared to using a memorized or no checklist was 0.47 (95%CI: 0.27, 0.83) for all mishaps (major and minor combined), and 0.31 (95% CI: 0.10, 0.93) for major mishaps. The rate of mishaps among memorized checklist users was similar to no checklist users.

**Conclusion:**

This study reinforces the utility of written checklists to prevent mishaps and, potentially, injuries and fatalities.

## Background

Recreational scuba diving is a sport in which divers use complex instruments and specialized skills in an unforgiving environment to survive and increase the duration of their underwater stay. The uncontrollable nature of the sport diving environment can lead to injuries and fatalities.

The incidence of all self-reported diving-related injuries was 3.1 per 1000 dives in a study of Divers Alert Network (DAN) members from the United States (Ranapurwala, Bird, Vaithiyanathan, & Denoble, 2014). DAN is a not-for-profit scuba diving membership organization that provides medical assistance, and education to divers, monitors diving injuries, and conducts dive medicine research. DAN researchers suggest that the number of recreational scuba diving fatalities in the United States and Canada varies between 80 and 100 per year (Denoble et al., 2008). The all-cause diving-related fatality rate in the United States and Canada has been estimated to be over 16/100,000 diver-years (Denoble et al., 2008). All-cause diving mortality studies from recreational and occupational divers suggest that most fatalities occur due to precursor events called incidents, errors, triggers or mishaps (Denoble, Caruso, Dear, Pieper, & Vann, 2008; Lippmann, 2010).

Mishaps are unwanted and unplanned events that occur during diving and increase the occurrence of injuries. They are precursors of injuries which may be contained given proper preparation and training. Diving mishaps result from human errors, equipment problems, or adverse environmental factors (Edmonds & Walker, 1989; Edmonds & Walker, 1990; Edmonds & Walker, 1991; Egstrom, 2004; Ranapurwala, 2014). Most common mishaps are running out-of-air, rapid ascent, equipment problems, and entrapment (Acott, 1999; Denoble et al., 2008). An extensive list of diving mishaps is published in the ‘diving incident report form,’ an ongoing self-reporting online survey by Divers Alert Network (DAN) Asia-Pacific (Acott, 1994). In this reporting system, 30% of the reported mishaps have been associated with injuries (Acott, 2003). In one study, the incidence of three mishaps, viz., low to out of gas, rapid ascent, and buoyancy problems - altogether, was 2.2/100 dives (Buzzacott, Denoble, Dunford, & Vann, 2009).

The mishaps are classified as major or minor mishaps, based on their potential to cause injury and the severity of those injuries. Major mishaps have a high potential to cause an injury, and the injury may be severe, or life-threatening, e.g., rapid ascent or running out-of-air. Minor mishaps have less potential to cause an injury, and the potential injuries are less severe, e.g., equalization problems or mask squeeze. Furthermore, there are conditions that exist prior to diving and may render diving unsafe. Recognition of unsafe conditions can lead a diver to take corrective measures. An example of such an unsafe condition would be unfamiliarity with equipment. If a diver dives with an unsafe condition the risk of a mishap and injury is increased.

Preventing diving mishaps may reduce the incidence of injuries and fatalities (Acott, 1994; Denoble et al., 2008), however there is very little information about the incidence of mishaps. Written checklists have been effectively used in other high risk areas like aviation and surgery to mitigate the risk of mishaps (Haynes et al., 2009; Helmreich, 2000), but the effectiveness of different forms of checklists (written or memorized) in diving is not known and the prevalence of checklist use among divers presumably low (Acott, 1995; Denoble et al., 2008; Ranapurwala et al., 2014).

A pre-dive checklist is a list of actions or tasks that allow a diver to check her equipment and readiness to dive in a stepwise manner. There is no gold standard pre-dive checklist in recreational scuba diving. Pre-dive checklists from different training agencies may differ considerably in content and practice. To make them easy to remember, training agencies develop mnemonics, e.g., BWRAF by Professional Association of Diving Instructors (PADI) (Professional Association of Diving Instructors, 2008), which stands for **B**uoyancy compensator device, **W**eights, **A**ir, **R**eleases, and **F**inal okay; or SEABAG by National Association of Underwater Instructors (NAUI) (National Association of Underwater Instructors, 2000), which stands for **S**ite survey, **E**mergency, **A**ctivity, **B**uoyancy, **A**ir, and **G**ear and go. However, using mnemonics may inadvertently reduce the use of written checklists among divers who choose to remember the mnemonic rather than carrying a checklist in print. In a study of recreational divers who were asked to perform equipment check procedures from memory (without using a written checklist), most were not be able to detect all the problems with the equipment (Acott, 1995). Some divers also make their own written pre-dive checklists. Thus, the actual use of checklists also varies in form and content.

This paper investigates the incidence of diving mishaps and unsafe conditions among recreational scuba divers and their association with divers’ use of their own written checklist.

## Methods

This was a nested study that utilized a prospective observational study design to evaluate the incidence of mishaps and unsafe conditions and their association with the routine use of divers own written checklists. The data for this study came from the control group of a cluster randomized trial (Trial Registration: ClinicalTrials.gov ID NCT01960738). The parent cluster randomized trial evaluated the effect of an investigator-developed pre-dive checklist on the incidence of diving mishaps among recreational scuba divers and four diving locations in Atlantic and Caribbean waters. The parent study has been described previously (Ranapurwala et al. 2016; Ranapurwala, 2014). This study follows the participants who did not receive the intervention checklist.

The data was collected from one location in North Carolina, two locations in Cozumel, Mexico, and one location in the Cayman Islands. Four trained personnel, one at each location, were placed and hosted by a total of seven dive shops. The study participants traveled together on dive boats operated by the participating dive shop, guided by the same boat operator, and made dives at the same locations.

A participant had to: be at least 18 years of age; have a valid diver certification; be planning to dive the day of their recruitment; be deemed fit for diving by the dive operator; and have working knowledge of English.

The divers were briefed about the study. Those agreeing to participate were asked to provide a signed consent. Participant names, contact information, age, sex, race and participant numbers were recorded during the consent. The participants were provided an outcomes questionnaire at the end of the participation day. The outcomes questionnaire asked divers about the number of dives they made that day, the mishaps and unsafe conditions that occurred during their dives (using a list of potential mishaps and unsafe conditions), information on height, weight, brief medical history, average annual dives, routine use of checklists (none/memorized/written), and information about the sea current (high/normal), visibility (poor/good), and animal attack (yes/no). The study personnel entered the data into an online data entry system.

The outcomes of interest were major mishaps, minor mishaps, all mishaps (major and minor combined), and unsafe conditions. These outcomes have been defined previously (Edmonds & Walker, 1989; Edmonds & Walker, 1990; Edmonds & Walker, 1991; Egstrom, 2004; Ranapurwala, 2014). The outcomes were coded as count data. In these analyses, the exposure was modeled in two ways. First, a three-category variable – no checklist, memorized checklist, and written checklist; and second, as a binary variable – written checklist versus memorized or no checklist.

This study was approved by the institutional review boards (IRB) at DAN # 009–12 on 03/27/2012 and the University of North Carolina (UNC) # 12–1051 on 05/29/2012, and subsequently renewed by IRB at DAN in 2013, 2014, 2015, 2016, and 2017. De-identified data can be made available by the authors upon request.

### Statistical methods

Poisson regression models were used to compare the incidence rates of mishaps and unsafe conditions among different checklist use groups. Since the participants on a location-day faced similar environmental conditions, their outcomes may not have been independent. To address the non-independence within a location-day cluster, generalized estimating equations were used (Hanley, Negassa, & Forrester, 2003).

The regression models were adjusted for demographic variables (age, sex and race) and potential confounders identified from a directed acyclic graph addressing the effect of checklists on the incidence of mishaps and unsafe conditions. The models were further reduced, if possible, by removing adjustment variables for which the squared change in the log rate ratio estimate (less adjusted minus more adjusted estimate) was smaller than the change in its estimated variance. Using this validity-precision tradeoff, the variable for the number of years a diver had been diving was dropped from the analysis.

The final models for evaluating the effect of checklist use on major mishaps and all mishaps included checklist use (binary or three-category exposure variable), sex (male/female), race (white/non-white), age (<35, ≥ 35), visibility (poor/good), current (high/normal), and average annual dives (continuous). The final models for minor mishaps and unsafe conditions excluded race due to non-convergence. Proportions of participants (crude) who chose to not dive were also calculated for written, memorized and no-checklist groups. SAS 9.3 (SAS Inc., Cary, N.C.) was used for all statistical analysis. Crude and adjusted rate ratios and 95% confidence intervals are reported.

## Results

Between June 1^st^ and August 17^th^ 2012, 467 divers were enrolled in the control group of the parent study. One participant withdrew, 10 were removed by the investigator because they did not complete the outcomes questionnaire, and 30 were lost-to-follow-up. The lost-to-follow-up occurred because of the difficulty of contacting every participant as they left the boats. Overall, 426 divers completed the study during 30 location-days. The average number of participants per location-day was 14.2 (range: 8–20), and the average number of dives per location-day was 28 (range: 15–42). Divers made a total of 840 dives ranging from 0 to 6 dives each; 22 divers chose not to dive (5.2%), 3.3% made one dive each, the majority (84.5%) made two dives each, and 7% made more than two dives each. More than two-thirds of the participants (n = 300) were males, the median age was 44 years (range 18–81 years), and the majority of the participants (96.2%) were White (Table 1). The median for average annual dives was 10 per diver (range 0 to 300). 8% of participants (n = 33) reported routinely using a self-checklist (written pre-dive checklist), 71% reported (n = 301) using memorized checklists, and 21% reported (n = 92) not using any kind of pre-dive checklist. High current while diving was reported by 78 participants (17%), and poor visibility was reported by 29 participants (6.8%).

**Table 1:** Covariate distribution among the self-checklist and no self-checklist group

Variables	Categories	Checklist types			
		Written		Memorized or none	
		N	%	N	%
Sex	Male	26	79	274	70
	Female	7	21	119	30
Age (in years)	18–35	4	12	117	30
	36 or more	29	88	276	70
Race	White	410	94	379	96
	Non-White^a^	2	6	14	4
Average yearly dives	0–20	20	61	278	70
	21–40	5	15	38	10
	>40	8	24	77	20
Bad visibility	Yes	6	18	23	6
	No	27	82	370	94
High current	Yes	3	9	73	19
	No	30	91	320	81
All participants		33 (7.8%)		393 (92.2%)	

^a^ includes African American, Asian, and Hispanic

About 36% of all participants (n = 153) reported either a major or minor mishap (range: 1–11 per diver). Rapid ascent and lost buddy contact were the most frequently observed major mishaps, and change in buoyancy due to dive suit, equalization problems, and mask problems were the most frequently observed minor mishaps (Table 2). The overall rate of major mishaps was 11.2 per 100 dives, the rate of minor mishaps was 18.2 per 100 dives and the rate of all the mishaps was 29.4 per 100 dives.

**Table 2:** Crude rates of mishaps in the written, memorized, and no-checklist group participants

	No. of mishaps	Crude rates per 100 dives		
		Written checklist (59 dives)	Memorized checklist (623 dives)	No checklist (158 dives)
Major mishaps	94	5.1	11.9	10.8
Rapid ascent	37	0.0	4.8	4.4
Lost buddy contact	22	3.4	2.7	1.6
Entanglement/ entrapment	9	0.0	1.1	1.3
Low to out of air	9	0.0	1.1	1.3
Free Ascent	8	0.0	1.1	0.6
Spontaneous inflation	5	0.0	0.8	0.0
Buddy breathing	2	1.7	0.0	0.6
2nd stage regulator malfunction	2	0.0	0.2	0.6
Content hose rupture	0	0.0	0.0	0.0
Free flowing 2nd stage	0	0.0	0.0	0.0
Minor mishaps	153	13.6	18.0	20.9
Changed buoyancy due to dive suit	29	3.4	3.0	5.1
Equalization problems	26	0.0	3.2	3.8
Mask squeeze	23	0.0	2.7	3.8
Mask flooded/ Dislodged/ Panic	17	1.7	2.4	0.6
O-ring problem	9	3.4	0.8	1.3
Buddy mismatch	7	1.7	0.8	0.6
Unable to clear mask	6	0.0	0.6	1.3
Fins lost/ loose/ dislodged	6	0.0	0.8	0.6
Safety Stop missed	6	0.0	0.8	0.6
Leaking BCD	6	1.7	0.8	0.0
Air used frequently to maintain buoyancy	5	0.0	0.3	1.9
Air not turned on/ not fully on	3	0.0	0.5	0.0
Computer stopped working	3	1.7	0.3	0.0
Weights belt/ weights dropped	2	0.0	0.3	0.0
Fins strap broke	2	0.0	0.2	0.6
Multiple ascents	2	0.0	0.3	0.0
Octopus reg snagged	1	0.0	0.0	0.6
Unable to release weights	0	0.0	0.0	0.0
Mask strap broke	0	0.0	0.0	0.0
All mishaps	247	18.6	29.9	31.6

The crude rate of major mishaps among the written checklist users was 5.1 per 100 dives compared to 11.9 and 10.8 per 100 dives in the memorized and no checklist groups (Table 2), a crude rate difference of 6.8 and 5.7 major mishaps per 100 dives, respectively. Similarly the crude rates of minor and all mishaps among routine written checklist users were also lower (Table 2).

At least one unsafe condition was reported by 18% (n = 80) of participants. One diver reported 8 unsafe conditions, while all others reported between 1 and 3. Most commonly reported unsafe conditions were being underweight, i.e., positively buoyant, and overweight, i.e., negatively buoyant in the water (Table 3). The overall crude rate of unsafe conditions was 12.9 per 100 dives. The crude rate of unsafe conditions for the written checklist users was 6.8 per 100 dives compared to 12.8 and 15.2 per 100 dives among the memorized and no checklist group (Table 3), a crude rate difference of 6.0 and 8.4 unsafe conditions per 100 dives, respectively. The proportion of divers who did not dive was 6.1% among the written checklist users compared to 2.3% among the memorized checklist users and 14.1% among the no checklist users.

**Table 3:** Crude rates of unsafe conditions in the written, memorized, and no checklist groups

	No. of unsafe conditions	Crude rate per 100 dives		
Outcome		Written checklist (59 dives)	Memorized checklist (623 dives)	No checklist (158 dives)
Underweight	26	3.4	2.7	4.4
Overweight	24	1.7	3.0	2.5
Not familiar with Dive computer	10	0.0	1.4	0.6
Tank not well secured	9	0.0	0.6	3.2
Unfamiliar with the use of BCD^a^	8	0.0	1.1	0.6
Tables Not used before	6	0.0	0.8	0.6
Tight/ uncomfortable dive suit	5	0.0	0.6	0.6
Dive computer battery problems	3	0.0	0.3	0.6
Incorrect BCD size	3	0.0	0.5	0.0
Size changed between dives	3	0.0	0.2	1.3
Fins - Caused cramp	2	0.0	0.3	0.0
Inaccurate dive gauge	2	1.7	0.0	0.6
BCD Inadequate buoyancy	2	0.0	0.3	0.0
Dive computer not turned on before dive	1	0.0	0.2	0.0
Dive tables – misread	1	0.0	0.2	0.0
Computer hard to understand	1	0.0	0.2	0.0
Unable to understand dive tables	1	0.0	0.2	0.0
Max depth indicator problem	1	0.0	0.2	0.0
Unable to locate alternate regulator	0	0.0	0.0	0.0
Unit confusion on the dive gauge	0	0.0	0.0	0.0
Total Unsafe conditions	108	6.8	12.8	15.2
		Proportion of divers (%)		
Participants who did not dive	22	6.1%	2.3%	14.1%

^a^includes confusion on BCD inflate/ deflate, & didn’t know how to inflate/ deflate the BCD

When memorized checklist use (referent) was compared with no checklist use, the adjusted rate ratio of major mishaps, minor mishaps, all mishaps, and unsafe conditions suggested that there was no difference between the memorized checklist group and no checklist group (Table 4). Hence the memorized checklist group and no checklist group were collapsed together and compared with the written checklist group (Table 4). With adjustment for covariates, written checklist users reported 69% fewer major mishaps (95% CI: 0.10, 0.93), 53% fewer total mishaps (95% CI: 0.27, 0.83), and 56% fewer unsafe conditions (95% CI: 0.22, 0.87) (Table 4). No injuries were observed in this study.

**Table 4:** Crude and adjusted rate ratios of mishaps and unsafe conditions comparing different checklist use groups

Mishaps	Rate ratios (95% Confidence Intervals)					
	No checklist vs memorized		Written vs memorized checklist		Written vs memorized or no checklist	
	Crude	Adjusted^a^	Crude	Adjusted^a^	Crude	Adjusted^a^
Major	0.85 (0.47, 1.53)	0.75 (0.43, 1.30)	0.42 (0.13, 1.37)	0.29 (0.10, 0.88)	0.43 (0.13, 1.41)	0.31 (0.10, 0.93)
Minor	1.06 (0.60, 1.87)	0.96 (0.56, 1.61)	0.65 (0.31, 1.34)	0.61 (0.29, 1.27)	0.64 (0.30, 1.35)	0.62 (0.29, 1.29)
All	0.97 (0.64, 1.48)	0.85 (0.59, 1.21)	0.55 (0.28, 1.09)	0.46 (0.26, 0.80)	0.55 (0.27, 1.13)	0.47 (0.27, 0.83)
Unsafe conditions	1.25 (0.72, 2.17)	1.16 (0.70, 1.95)	0.55 (0.30, 1.02)	0.45 (0.22, 0.92)	0.52 (0.28, 0.98)	0.44 (0.22, 0.87)

^a^Adjusted for sex, race, age (<35 years/ ≥ 35 years), poor-visibility, high-current, and average annual dives

## Discussion

In our study divers who reported routinely using a written checklist (provided by their training agencies or self-made) had lower rates of mishaps and unsafe conditions than divers who did not use a written checklist. Major mishap rates were lower by more than two-thirds, all mishaps combined were lower by more than a half, and unsafe conditions were also lower by more than a half. Only eight percent of participants in the study reported routinely using a written pre-dive checklist, suggesting a great potential for increased use of written checklists to reduce injuries among recreational scuba divers. It is possible that any well-designed written checklist would yield benefits. The efficacy of written checklists in recreational scuba diving is supported by their efficacy in other high risks areas like aviation and surgery (Haynes et al., 2009; Helmreich, 2000).

Among the major mishaps, rapid ascent and lost buddy contact were most common. Rapid ascent may be related to an acute health event, equipment failure, running out of air, panicking during the dive, or inattention to ascent rate (Buzzacott, Rosenberg, & Pikora, 2009). The last three possible explanations suggest inexperience or lack of proper training. Lost buddy contact could result from similar conditions or environmental conditions like lack of visibility or strong current. The conventional rule in the case of lost buddy contact is to search for the buddy for one minute and if not found, start a controlled ascent. Although this rule is taught by diving agencies, compliance is questionable in part because early ascent prevents divers from using all the dive time they have purchased. Lost buddy contact is one of the most frequently encountered features in diving-related fatalities (Denoble et al., 2008; Walker, Lippmann, Lawrence, Houston, & Fock, 2009). The common occurrence of such avoidable mishaps suggest that using written checklist can help a diver be better prepared.

Entanglement/ entrapment and low-to-out-of-air were the next most common major mishaps. All-cause diving-related mortality studies suggest that insufficient gas and entrapment are the leading triggers in diving fatalities (Denoble et al., 2008). Despite these major mishaps, no injuries were observed in this study. Many individuals carried inappropriate weights and were underweight (positively buoyant) or overweight (negatively buoyant) (Table 3) which may lead to overexerting while going underwater or resurfacing potentially leading to low-to-out-of-air situations; this also reflects inexperience or lack of training.

Participants reported more minor mishaps than unsafe conditions (Tables 2 and 3). Conventionally, the contrary would be expected because unsafe conditions are precursors of mishaps, however not all unsafe conditions lead to mishaps and some mishaps may even occur in the absence of an unsafe condition on the diver’s part. The possible explanation may lie in the nature of the problem. Unsafe conditions can be identified and often corrected easily before the dive starts. The diver may have corrected some of the unsafe conditions and hence not reported them, leading to fewer reported unsafe conditions overall. In fact, written checklist users may have reported fewer unsafe conditions solely because they identified the unsafe conditions using their written checklists even before the dive began, and rectified them.

Our results also suggest that memorized checklists are not any better than not having a checklist. Since most of the memorized diving checklists utilize mnemonics, it may be inferred that mnemonics do not provide focused instructions, and divers may only remember the mnemonic and the broad categories the mnemonics represent, but forget or overlook the specific actions to be taken. For example, divers may remember the mnemonic BWRAF as Buoyancy, Weights, Releases, Air, and Final-ok, but may forget the checks to be performed under each of these headings. Our findings are consistent with a 1995 study, where 55 recreational scuba divers who attended a dive equipment exhibition were asked to perform a pre-dive check on a pre-assembled scuba gear from their memory and experience without using a written checklist. The researchers had deliberately left nine equipment faults for the divers to find. Only 2 out of 55 divers found all nine faults (Acott, 1995).

This study has several limitations. Because it was conducted in the Atlantic and Caribbean waters during summer 2012, where current, visibility, water temperature, and wild animal populations differ from other regions and time periods, the results may not be generalizable to all diving conditions. Additionally, checklist use among recreational scuba divers may have a different impact than use among technical divers, seafood harvesters, or navy divers. Furthermore, the effect of written checklist use may depend on the contents of the checklist; checklists used by participants in this study were not evaluated and may have varied greatly. However, pre-dive checklists that conform to diving safety guidelines should not have any negative impact, and therefore should be safe to use in any environment, season, or type of diving.

## Conclusions

Our study suggests that the incidence of recreational diving mishaps is substantial and the prevalence of routine use of written checklists is low. The routine use of a written pre-dive checklist, irrespective of its source and content, was associated with fewer mishaps and unsafe conditions during the specific dives under study. The results of our study suggests that routine use of written pre-dive checklists is an effective tool for promoting diving safety. The use of memorized checklists was similar to not using any checklist at all; hence the use of written checklists should be encouraged, instead.

Future studies may examine factors that promote or discourage checklist use and divers’ perceptions about pre-dive checklists. Exploring these areas will help develop targeted methods to promote checklist use and a culture of safety, educate divers, and reduce the incidence of injuries and fatalities.

## Abbreviations

BCD: buoyancy control device
CI: confidence interval
